# Competition in Coordination Assemblies: 1D-Coordination Polymer or 2D-Nets Based on Co(NCS)_2_ and 4′-(4-methoxyphenyl)-3,2′:6′,3″-terpyridine

**DOI:** 10.3390/polym11071224

**Published:** 2019-07-23

**Authors:** Dalila Rocco, Alessandro Prescimone, Y. Maximilian Klein, Dariusz J. Gawryluk, Edwin C. Constable, Catherine E. Housecroft

**Affiliations:** 1 Department of Chemistry, University of Basel, BPR 1096, Mattenstrasse 24a, CH-4058 Basel, Switzerland; 2 Laboratory for Multiscale Materials Experiments, Paul Scherrer Institut, CH-5232 Villigen PSI, Switzerland

**Keywords:** 3,2′:6′,3″-terpyridine, coordination polymer, coordination network, cobalt(II) thiocyanate

## Abstract

The synthesis and characterization of 4′-(4-methoxyphenyl)-3,2′:6′,3″-terpyridine (**2**) (IUPAC PIN 2^4^-(4-methoxyphenyl)-1^2^,2^2^:2^6^,3^2^-terpyridine) are described, and its coordination behaviour with cobalt(II) thiocyanate has been investigated. In a series of experiments, crystals were grown at room temperature by layering a MeOH solution of Co(NCS)_2_ over a CHCl_3_ solution of **2** using 1:1, 1:2 or 2:1 molar ratios of metal salt-to-ligand. Crystals harvested within 2–3 weeks proved to be the 1D-coordination polymer [Co(**2**)(NCS)_2_(MeOH)_2_]*_n_* and powder X-ray diffraction (PXRD) confirmed that the crystals selected for single-crystal X-ray diffraction were representative of the bulk samples. Longer crystallization times with a Co(NCS)_2_ to **2** molar ratio of 1:1 yielded crystals of [Co(**2**)(NCS)_2_(MeOH)_2_]*_n_* (1D-chain) and the pseudopolymorphs [{Co(**2**)_2_(NCS)_2_}·3MeOH]*_n_* and [{Co(**2**)_2_(NCS)_2_}·2.2CHCl_3_]*_n_* ((4,4)-nets), each type of crystal originating from a different zone in the crystallization tube. PXRD for this last experiment confirmed that the dominant product in the bulk sample was the 1D-coordination polymer.

## 1. Introduction

The use of vectorially preorganized building blocks for the rational design of 2- and 3-dimensional metal coordination architectures is a well-developed field [1,2], and leads to the tailored assembly of metal-organic frameworks (MOFs) with widespread applications [3] including gas storage [4,5], separation [6], photocatalysis [7], drug delivery [8,9,10], and water purification [11,12]. A key factor in controlling MOF assembly is the use of rigid organic linkers such as 4,4′-bipyridine. However, the conflict between achieving a predetermined or a serendipitous assembly is an underlying problem for the coordination chemist or crystal engineer. Shimizu has illustrated that there is scope for developing metal coordination assemblies with more flexible ligands, proposing that a ligand with “less well-defined properties does not necessarily translate to less functional materials” [13] and we have described similar self-assembly processes as examples of fuzzy logic [14].

**Scheme 1:**
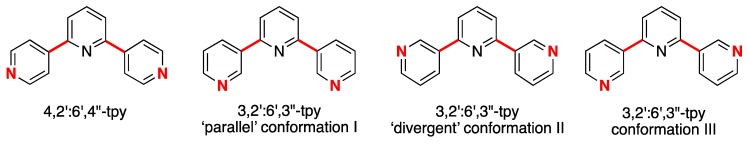
Structures of the isomers 4,2′:6′,4″- and 3,2′:6′,3″-terpyridine (tpy), showing examples of the different conformations of 3,2′:6′,3″-tpy. Coordination of metal ions occurs through the outer *N*-donors (shown in red). See text for the significance of the C–C bonds marked in red.

We have been focusing on metal coordination assemblies based on two isomers of terpyridine, 4,2′:6′,4″- and 3,2′:6′,3″-terpyridine (IUPAC PIN 1^4^,2^2^:2^6^,3^4^- and 1^3^,2^2^:2^6^,3^3^-terpyridine [15]) (Scheme 1) [16,17,18,19]. The nitrogen atom of the central pyridine ring is not involved in metal coordination and, therefore, these ligands are ditopic [18]. As we have previously discussed [17,18], rotation about the inter-ring C–C bonds (Scheme 1) maintains a constant vectorial relationship of the two nitrogen donors in the V-shaped 4,2′:6′,4″-tpy shown in Scheme 1. In contrast, the vectorial arrangement of the two nitrogen atoms marked in red in 3,2′:6′,3″-tpy in Scheme 1 is dependent upon conformational changes in the ligand. Functionalization of the tpy unit in the 4′-position is easily achieved using the one-pot method of Wang and Hanan [20], and we have applied this strategy to investigate the effects on metal coordination assemblies of introducing a range of substituents into 4,2′:6′,4″-tpy on metal coordination assemblies. Systematic studies in which only one parameter is varied at one time provide the most valuable insight into design strategies to direct coordination architectures. However, such investigations are relatively scarce in the literature (for example, [21,22]), and analysis of data from different research groups is often complicated by use of different solvents and crystallization methods. The fact that two or more metalloassemblies (e.g., metallocycles, metallopolymers) may be close in energy is the underlying principle for the development of libraries of architectures (for example, [23,24,25,26,27]).

**Scheme 2:**
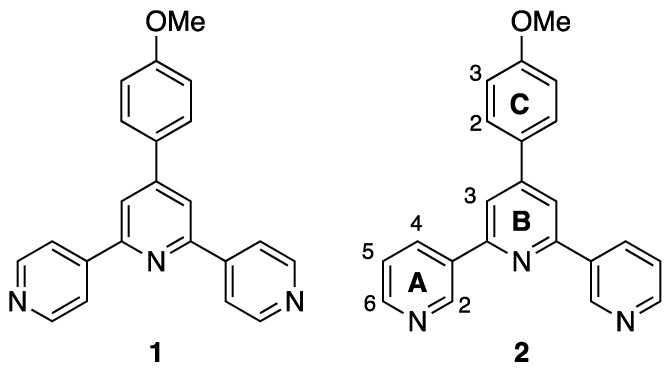
Structures of ligands **1** and **2** with atom numbering scheme for the NMR assignments of **2**.

We have previously investigated the coordination behaviour of 4′-(4-methoxyphenyl)-4,2′:6′,4″-terpyridine, **1**, (Scheme 2). A 1D-coordination polymer [Zn_2_(μ-OAc)_4_(**1**)]*_n_* formed under room temperature conditions of layering an MeOH solution of Zn(OAc)_2_·2H_2_O over a CHCl_3_ solution of **1** [21]. The assembly of a 1D-chain arises from the linear disposition of the vacant axial coordination sites in a paddle-wheel {Zn_2_(μ-OAc)_4_} unit [2,28], and from the input molar ratio of Zn(OAc)_2_ to **1** which was 2:1 [21]. Using similar layering conditions at ambient temperatures, reaction of **1** with cadmium(II) nitrate led to the assembly of a ladder [{Cd_2_(**1**)_3_(NO_3_)_4_}·CHCl_3_·MeOH]*_n_* in which the ligand **1** acts as a linker, forming both the “rungs” and “rails” of the ladder. The 2:3 Cd to **1** ratio in the structure contrasted with the input molar ratio of 3:1, but analysis of other crystals from separate crystallization tubes confirmed this stoichiometry for the crystalline coordination polymer [29]. In directing assemblies, cobalt(II) thiocyanate is a potential 4-connecting node, i.e., octahedral coordination with two sites occupied by thiocyanato ligands. Therefore, the observed assembly of a (4,4) net from a combination of **1** and Co(NCS)_2_ is both readily understood and expected. The 2D-net [{Co_2_(NCS)_4_(**1**)_4_}·2CHCl_3_·1.5MeOH]*_n_* was obtained under conditions of layering an MeOH solution of Co(NCS)_2_ over a CHCl_3_ solution of **1**, even though the molar input ratio of Co:**1** was 1:3 [30]. A similar (4,4) net, [{Co(**1**)_2_(NCS)_2_}·2MeCN·2DMF]*_n_* (DMF = dimethylformamide), has been reported by Konar and co-workers, although in this case, hydrothermal conditions were used with an input molar ratio of Co to **1** of 2:1 [31]. Therefore, in both cases, irrespective of crystallization method, there is a preference for a (4,4) net with a Co to **1** ratio of 1:2. An analogous (4,4) net is found in [{Fe(**1**)_2_(NCS)_2_}·2MeCN]*_n_*, again formed hydrothermal conditions [31]. Based on a search of the Cambridge Structural Database (CSD, v. 5.40 with February 2019 updates [32], using Conquest v. 2.0.1 [33]), the only other reports of structurally characterized coordination networks involving ligand **1** describe reactions with copper(I) iodide under hydrothermal synthetic conditions to give a series of related (4,4) nets with 4-connecting {Cu_2_I_2_} nodes [34,35]. The intriguing difference between these 2D-networks and those based on Co(NCS)_2_ is that the copper(I)-containing assemblies are interpenetrating, leading to porous 3D-frameworks which are thermally robust and can undergo reversible solvent exchange [34].

Noting the observations discussed above, we decided to carry out an investigation of the reaction of ligand **2** (Scheme 2) with Co(NCS)_2_, with the aim of comparing the coordination of the isomeric ligands **1** and **2** with Co(NCS)_2_, and to gain a better understanding of the effects of varying the metal to ligand ratio in the crystallization tubes. 

## 2. Materials and Methods

### 2.1. General

^1^H and ^13^C{^1^H} NMR spectra were recorded on a Bruker Avance iii-500 spectrometer (Bruker BioSpin AG, Fällanden, Switzerland) at 298 K. The ^1^H and ^13^C NMR chemical shifts were referenced with respect to residual solvent peaks (*δ* TMS = 0). Electrospray ionization (ESI) mass spectra were recorded using a Shimadzu LCMS-2020 instrument (Shimadzu Schweiz GmbH, Reinach, Switzerland) samples were introduced as 200–800 μM solutions in MeCN with the addition of formic acid. PerkinElmer UATR Two (Perkin Elmer, Schwerzenbach, Switzerland) and Cary-5000 (Agilent Technologies Inc., Santa Clara, CA, USA) spectrometers were used to record FT-infrared (IR) and absorption spectra, respectively.

3-Acetylpyridine was purchased from Acros Organics (Chemie Brunschwig AG, Basel, Switzerland), and 4-methoxybenzaldehyde and Co(NCS)_2_ were bought from Sigma Aldrich (Steinheim, Germany). All chemicals were used as received. 

### 2.2. Synthesis of **2**

4-Methoxybenzaldehyde (1.36 g, 1.22 mL, 10.0 mmol) was dissolved in EtOH (50 mL), and then 3-acetylpyridine (2.42 g, 2.20 mL, 20.0 mmol) and crushed KOH (1.12 g, 20.0 mmol) were added, upon which the solution changed from colourless to yellow. Aqueous NH_3_ (32%, 38.5 mL) was slowly added to the reaction mixture. This was stirred at room temperature overnight. The solid that formed was collected by filtration, washed with water (3 × 10 mL), EtOH (3 × 10 mL), recrystallized from EtOH and dried in vacuo. Compound **2** was isolated as a white powder (0.888 g, 2.62 mmol, 26.2%). M.p. = 157 °C. ^1^H NMR (500 MHz, CDCl_3_): *δ*/ppm = 9.38 (d, *J* = 2.1 Hz, 2H, H^A2^), 8.71 (dd, *J* = 4.8, 1.5 Hz, 2H, H^A6^), 8.55 (m, 2H, H^A4^), 7.94 (s, 2H, H^B3^), 7.72 (m, 2H, H^C2^), 7.50 (m, 2H, H^A5^), 7.08 (m, 2H, H^C3^), 3.90 (s, 3H, H^a^). ^13^C{^1^H} NMR (500 MHz, CDCl_3_): *δ*/ppm = 161.1 (C^C4^), 155.2 (C^A3^), 150.7 (C^B4^), 149.6 (C^A6^), 147.9 (C^A2^), 135.3 (C^A4^), 135.2 (C^B2^), 130.4 (C^C1^), 128.5 (C^C2^), 124.0 (C^A5^), 117.5 (C^B3^), 114.9 (C^C3^), 55.6 (C^a^). UV–VIS (CH_3_CN, 5.0 × 10^−5^ mol dm^−3^) λ/nm 228 (ε/dm^3^ mol^−1^ cm^−1^ 24,400), 274 (30,000). ESI-MS *m/z* 340.10 [M + H]^+^ (calc. 340.14). Found C 77.74, H 5.05, N 12.36; required for C_22_H_17_N_3_O: C 77.86, H 5.05, N 12.38.

### 2.3. [Co(2)(NCS)_2_(MeOH)_2_]_n_, [{Co(2)_2_(NCS)_2_}·3MeOH]_n_, and [{Co(2)_2_(NCS)_2_}·2.2CHCl_3_]_n_

In Experiment I, a solution of Co(NCS)_2_ (5.3 mg, 0.030 mmol) in MeOH (6 mL) was layered over a CHCl_3_ solution (4 mL) of **2** (10.2 mg, 0.030 mmol). Pink block-like crystals visible to the eye were first obtained after 17 days and a single crystal was selected for X-ray diffraction after another 4 weeks. This proved to be [Co(**2**)(NCS)_2_(MeOH)_2_]*_n_*. Cell dimension checks carried out on two other block-shaped crystals from the crystallization tube confirmed consistent cell dimensions. The remaining crystals in the tube were washed with MeOH and then CHCl_3_, dried under vacuum and were analysed by powder X-ray diffraction (PXRD). 

In Experiment II, a solution of Co(NCS)_2_ (5.3 mg, 0.030 mmol) in MeOH (6 mL) was layered over a CHCl_3_ solution (4 mL) of **2** (10.2 mg, 0.030 mmol). Pink crystals visible to the eye started growing after one week and a single crystal selected for X-ray diffraction after a further week was found to be [{Co(**2**)_2_(NCS)_2_}·3MeOH]*_n_*. A second crystal was removed from the bottom of the tube after a further 5 weeks and single-crystal X-ray diffraction confirmed this to be [{Co(**2**)_2_(NCS)_2_}·2.2CHCl_3_]*_n_*. A cell dimension check was carried out on one of the block-like crystals growing on the wall of the tube confirmed it to be [Co(**2**)(NCS)_2_(MeOH)_2_]*_n_*. The remaining crystals were washed with MeOH and CHCl_3_, dried under vacuum and were analysed by PXRD.

In Experiment III, a solution of Co(NCS)_2_ (10.5 mg, 0.0600 mmol) in MeOH (6 mL) was layered over a CHCl_3_ solution (4 mL) of **2** (10.2 mg, 0.030 mmol). Pink blocks visible to the eye started growing after 17 days and the tube was left for 3 months at room temperature. A cell dimension check on one crystal from this tube was consistent with [Co(**2**)(NCS)_2_(MeOH)_2_]*_n_*_._ The remaining crystals in the tube were washed with MeOH and CHCl_3_, dried under vacuum and were analysed by PXRD. 

In Experiment IV, a solution of Co(NCS)_2_ (2.6 mg, 0.015 mmol) in MeOH (5 mL) was layered over a CHCl_3_ solution (5 mL) of **2** (10.2 mg, 0.030 mmol). A few pink plates visible to the eye started growing after 15 days at room temperature. A cell dimension check on one crystal from this tube was consistent with [Co(**2**)(NCS)_2_(MeOH)_2_]*_n_*. Insufficient amount of material was available for PXRD.

Bulk reactions were carried out with 1:1 and 1:2 Co(NCS)_2_ to **2** molar ratios. Solid Co(NCS)_2_ (51.7 mg, 0.295 mmol) was added to a colourless solution of **2** (100 mg, 0.295 mmol) in MeOH (40 mL). Immediate precipitation of a pale pink solid was observed and the mixture was left stirring at room temperature for 15 h. The product was separated by centrifuge followed by decantation of the supernatant liquid. The solid was washed with MeOH (2 × 10 mL) and with CHCl_3_ (10 mL) to remove unreacted reagents and was then dried under vacuum. The procedure for the 1:2 reaction was the same, starting with Co(NCS)_2_ (25.7 mg, 0.147 mmol) and **2** (100 mg, 0.295 mmol). The two solid products were investigated by PXRD.

### 2.4. Crystallography

Single crystal data for [{Co(**2**)_2_(NCS)_2_}·3MeOH]*_n_* and [{Co(**2**)_2_(NCS)_2_}·2.2CHCl_3_]*_n_* were collected on a Bruker APEX-II diffractometer with data reduction, solution and refinement using the programs APEX [36], ShelXT [37], Olex2 [38] and ShelXL v. 2014/7 [39]. For [Co(**2**)(NCS)_2_(MeOH)_2_]*_n_*, single crystal data were collected on a STOE StadiVari diffractometer equipped with a Pilatus300K detector and with a Metaljet D2 source; the structure was solved using Superflip [40,41] and Olex2 [38]. The model was refined with ShelXL v. 2014/7 [39]. Structure analysis used Mercury CSD v. 4.1.0 [42,43]. 

SQUEEZE [44] was used in [{Co(**2**)_2_(NCS)_2_}·3MeOH]*_n_*. The electrons removed equated to 3 molecules of MeOH per cobalt atom. 

Powder X-Ray Diffraction (PXRD) patterns were collected at room temperature in the Debye-Scherrer geometry using a Bruker AXS D8 Advance diffractometer (Bruker AXS GmbH, Karlsruhe, Germany) equipped with a Ni-filtered Cu Kα radiation and a 1D LynxEye PSD detector. The reflections of a main phase were indexed with an orthorhombic cell in the space group *Pbca* (No 61). The Rietveld refinement analysis [45] of the diffraction patterns was performed with the package FULLPROF SUITE [46,47] (version March-2019) using a previously determined instrument resolution function (based on the small line width polycrystalline sample Na_2_Ca_3_Al_2_F_14_ measurements [48,49]). The structural model was taken from the single-crystal X-Ray diffraction refinement. Refined parameters were: scale factor, zero displacement, lattice parameters, Co and S atomic positions, and peak shapes as a Thompson–Cox–Hastings pseudo-Voigt function. Due to the powdered crystals’ habit, a preferred orientation as a March–Dollase multi-axial phenomenological model was implemented in the analysis.

### 2.5. [Co(2)(NCS)_2_(MeOH)_2_]_n_

C_26_H_25_CoN_5_O_3_S_2_, *M_r_* = 578.56, pink block, orthorhombic, space group *Pbca*, *a* = 14.0281(3), *b* = 14.3649(3), *c* = 26.0077(6) Å, *V* = 5240.9(2) Å^3^, *D_c_* = 1.467 g cm^−3^, *T* = 130 K, *Z* = 8, *Z’* = 1, *μ*(GaK*α*) = 4.713 mm^−1^. Total 98166 reflections, 5359 unique (*R_int_* = 0.3579). Refinement of 3094 reflections (343 parameters) with *I* > 2*σ*(*I*) converged at final *R*_1_ = 0.0639 (*R*_1_ all data = 0.1341), *wR*_2_ = 0.1376 (*wR*2 all data = 0.1728), gof = 1.006. CCDC 1922228.

### 2.6. [{Co(2)_2_(NCS)_2_}·3MeOH]_n_

C_49_H_46_CoN_8_O_5_S_2_, *M_r_* = 949.99, pink plate, monoclinic, space group *P*2_1_/*n*, *a* = 13.2570(6), *b* = 12.1980(5), *c* = 15.0937(6) Å, *β* = 98.675(2), *V* = 2412.86(18) Å^3^, *D_c_* = 1.308 g cm^−3^, *T* = 130 K, *Z* = 2, *Z’* = 0.5, *μ*(CuK*α*) = 4.069 mm^−1^. Total 15819 reflections, 4303 unique (*R_int_* = 0.0336). Refinement of 3963 reflections (269 parameters) with *I* > 2*σ*(*I*) converged at final *R*_1_ = 0.0386 (*R*_1_ all data = 0.0420), *wR*_2_ = 0.1092 (*wR*2 all data = 0.1118), gof = 1.062. CCDC 1922226.

### 2.7. [{Co(2)_2_(NCS)_2_}·2.2CHCl_3_]_n_

C_48.2_H_36.6_Cl_6.6_CoN_8_O_2_S_2_, *M*_r_ = 1116.87, pink block, monoclinic, space group *P*2_1_/*c*, *a* = 11.0555(8), *b* = 13.2414(10), *c* = 18.5561(13) Å, *β* = 96.956(3), *V* = 2696.4(3) Å^3^, *D*_c_ = 1.376 g cm^−3^, *T* = 130 K, *Z* = 2, *Z*’ = 0.5, *μ*(CuK*α*)= 6.592 mm^−1^. Total 23049 reflections, 4886 unique (*R*_int_ = 0.0313). Refinement of 4652 reflections (341 parameters) with *I* > 2σ*(I*) converged at final *R*_1_ = 0.1177 (*R*_1_ all data = 0.1199), *wR*_2_ = 0.4012 (*wR*_2_ all data = 0.4131), gof = 1.096. CCDC 1922227.

## 3. Results and Discussion

### 3.1. Ligand Synthesis and Characterization

**Figure 1:**
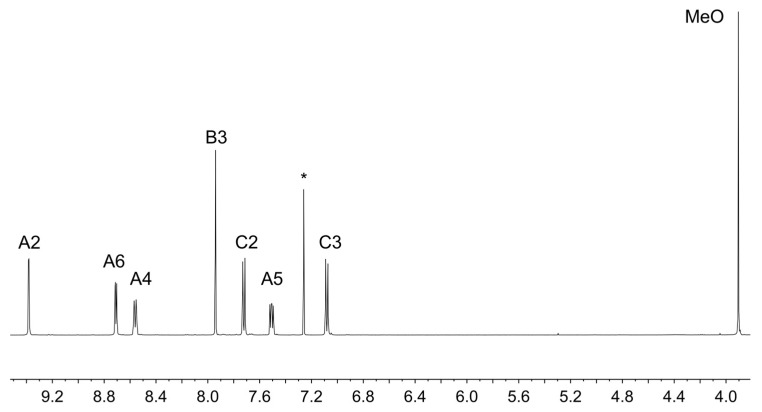
500 ^1^H MHz NMR spectrum of **2** (CDCl_3_, 298 K). * = residual CHCl_3_. See Scheme 2 for atom labelling.

**Figure 2:**
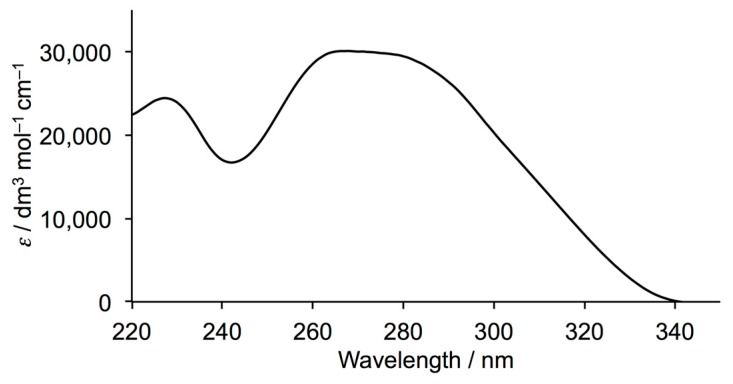
Solution absorption spectrum of compound **2** (MeCN, 5 × 10^−5^ mol dm^−3^).

Compound **2** was prepared by Hanan’s [20] one-pot synthesis from 3-acetylpyridine and 4-methoxybenzaldehyde in the presence of base, followed by the addition of aqueous ammonia to give **2** in 26.2% yield after purification. A single peak in the electrospray mass spectrum at *m/z* = 340.10 (Figure S1, see Supporting Information) corresponded to [M + H]^+^. The ^1^H and ^13^C{^1^H} NMR spectra were assigned using 2D-methods. Figure 1 shows the ^1^H NMR spectrum and the ^13^C{^1^H} NMR, NOESY, HMQC and HMBC spectra are shown in Figures S2–S5 (see Scheme 2 for atom labelling). The spectra are consistent with a time-averaged C_2_-symmetric ligand and protons H^C2^ and H^C3^ were distinguished by the NOESY crosspeak between H^B3^ and H^C2^ (Figure S3). The solution absorption spectrum of **2** (Figure 2) exhibits intense absorptions arising from spin-allowed π*←*n* and π*←π transitions, and is comparable to the absorption spectra of the analogous ligands bearing ethoxy or *n*-butoxy substituents [50].

### 3.2. Reactions of Compound **2** with Co(NCS)_2_

**Scheme 3:**
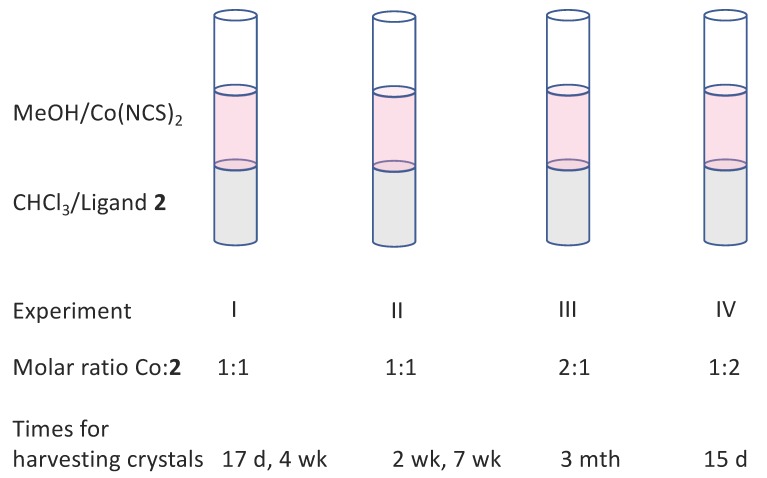
Summary of Experiments I–IV.

Four tubes, each containing a MeOH solution of Co(NCS)_2_ layered over a CHCl_3_ solution of **2**, were left standing at room temperature (ca. 295 K). In two of the tubes (Experiments I and II), identical conditions were used with a molar ratio of Co(NCS)_2_:**2** of 1:1. In Experiments III and IV, the same crystallization conditions were used as in Experiments I and II but with molar ratios of Co(NCS)_2_:**2** of 2:1 and 1:2, respectively. The experiments are summarized in Scheme 3 and details of the rate of crystal growth and the selection of crystals are given in the Materials and Methods Section 2.3. 

In Experiments I and III, pink block-like single crystals of [Co(**2**)(NCS)_2_(MeOH)_2_]*_n_* were obtained, while in Tube II, three different crystal types were found in different zones in the tube. Blocks of [Co(**2**)(NCS)_2_(MeOH)_2_]*_n_* grew on the walls of the tube. Pink block-like crystals of [{Co(**2**)_2_(NCS)_2_}·2.2CHCl_3_]*_n_* formed at the bottom of the tube and it is relevant that initially, the CHCl_3_ solution of the ligand comprised the lower layer in the crystallization tube. Higher up on the walls of the tube, pink plates of [{Co(**2**)_2_(NCS)_2_}·3MeOH]*_n_* were obtained, consistent with growth in a zone where the concentration of methanol exceeds that of chloroform. As described below, [{Co(**2**)_2_(NCS)_2_}·2.2CHCl_3_]*_n_* and [{Co(**2**)_2_(NCS)_2_}·3MeOH]*_n_* are pseudopolymorphs, differing only in the inclusion of different amounts of different lattice solvent [51,52]. 

**Figure 3:**
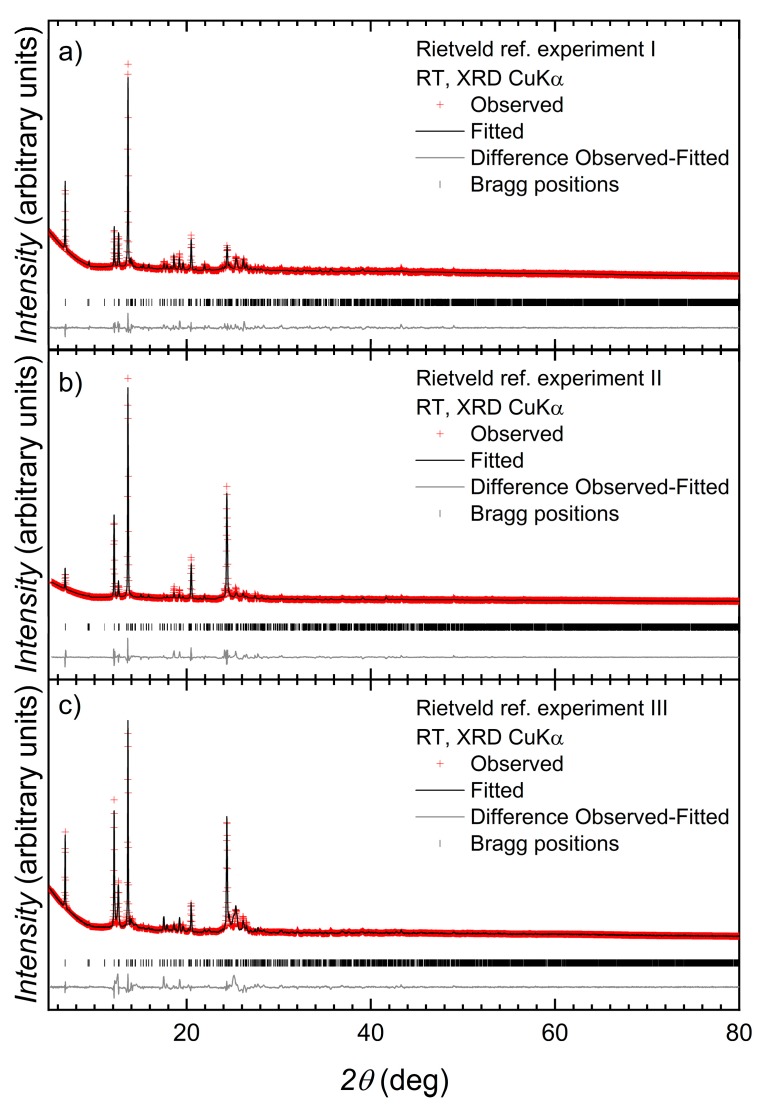
Laboratory X-ray diffraction (CuKα radiation) pattern (red crosses) for Experiments (**a**) I, (**b**) II, and (**c**) III (space group *Pbca*) at room temperature. The black line corresponds to the best fit from the Rietveld refinement analysis. Lower vertical marks denote the Bragg peak positions. The bottom, grey line represents the difference between experimental and calculated points.

The PXRD patterns of the bulk material from Experiments I and III are displayed in Figures S6 and S7 and a comparison with the pattern predicted from the single crystal structure of [Co(**2**)(NCS)_2_(MeOH)_2_]*_n_* (see below) indicates that the single crystal selected was representative of all single crystals that grew from the 1:1 and 2:1, respectively, reactions of Co(NCS)_2_ with **2**. A Rietveld refinement of the diffraction patterns, using the structural model from the single crystal refinement, was carried out using FULLPROF SUITE [46,47]. The refinement confirmed, that the bulk sample from each of Experiments I and III is [Co(**2**)(NCS)_2_(MeOH)_2_]*_n_* (Figure 3a,c). Preferred orientations in this material correspond to the (001) and (010) lattice planes. A bulk reaction with a 1:1 molar ratio of reactants was also carried out (see Materials and Methods Section). Although the sample was of low crystallinity, peaks at low values of 2*θ* (in particular that at 2*θ* = 6.82°) in the PXRD pattern (Figure S8) were consistent with [Co(**2**)(NCS)_2_(MeOH)_2_]*_n_*.

Figure S9 shows a comparison of the PXRD pattern for the bulk material from Experiment II with those predicted from the singles crystal structures of [Co(**2**)(NCS)_2_(MeOH)_2_]*_n_*, [{Co(**2**)_2_(NCS)_2_}·3MeOH]*_n_* and [{Co(**2**)_2_(NCS)_2_}·2.2CHCl_3_]*_n_*. The Rietveld refinement confirmed that the bulk sample represents the 1D coordination polymer (Figure 3b). Very small amounts of other species were observed in the PXRD, which could not be assigned to [{Co(**2**)_2_(NCS)_2_}·3MeOH]*_n_* or [{Co(**2**)_2_(NCS)_2_}·2.2CHCl_3_]*_n_*.

### 3.3. Structure of [Co(2)(NCS)_2_(MeOH)_2_]_n_

**Figure 4:**
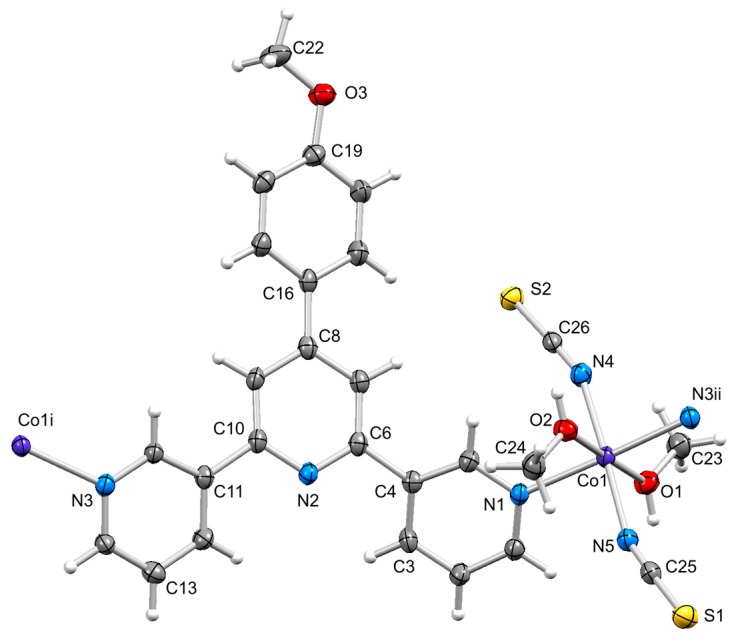
The repeating unit in the 1D chain of [Co(**2**)(NCS)_2_(MeOH)_2_]*_n_* with symmetry generated atoms; ellipsoids are plotted at a 40% probability level. Symmetry codes: i = ^1^/_2_–*x*, 1–*y*, –^1^/_2_+*z*; ii = ^1^/_2_–*x*, 1–*y*, ^1^/_2_+*z*. Selected bond distances and angles: Co1–O2 = 2.109(4), Co1–O1 = 2.084(4), Co1–N1 2.184(4), Co1–N5 = 2.073(4), Co1–N4 2.084(4), Co1–N3^ii^ = 2.196(4), O3–C19 = 1.362(6), O3–C22 = 1.422(7), O2–C24 1.439(7), O1–C23 = 1.417(7) Å; N5–Co1–N4 = 178.00(16), N1–Co1–N3^ii^ = 176.33(17), O1–Co1–O2 = 179.14(14), C24–O2–Co1 = 128.9(3), C23–O1–Co1 = 127.7(3), C19–O3–C22 = 117.7(4)°.

[Co(**2**)(NCS)_2_(MeOH)_2_]*_n_* crystallizes in the orthorhombic space group *Pbca*. The structure is closely related to that of [Co(**3**)(NCS)_2_(MeOH)_2_]*_n_* in which ligand **3** is 4′-(*N,N-*dimethylaminophenyl)-3,2′:6′,3″-terpyridine (CSD refcode ZAWLUG, space group *Pbca*, *a* = 14.3014(8), *b* = 14.3112(7), *c* = 26.2091(12) Å) [53]. The cobalt(II) centre in [Co(**2**)(NCS)_2_(MeOH)_2_]*_n_* is six-coordinate with *trans*-arrangements of pairs of ligands (Figure 4). Ligand **2** coordinates through the outer pyridine rings to two different cobalt atoms and the repeating unit shown in Figure 4 propagates into a 1D-coordination polymer chain. Important bond distances and angles are given in the caption to Figure 4 and are unexceptional. The outer pyridine rings of ligand **2** are oriented so as to provide a building block similar to Conformation II in Scheme 1. Rotation about the C–C inter-ring bonds shown in red in Scheme 1 would allow ligand **2** to adopt an infinite number of rotational conformations. The 3,2′:6′,3″-tpy unit is twisted with angles of 35.2° and 21.2° between the least squares planes of the rings containing N1/N2 and N2/N3, respectively. In contrast, the central 4′-phenylpyridine unit deviates less from planarity with an angle of only 12.4° between the ring planes. This is associated with packing interactions between the chains (see below).

**Figure 5:**
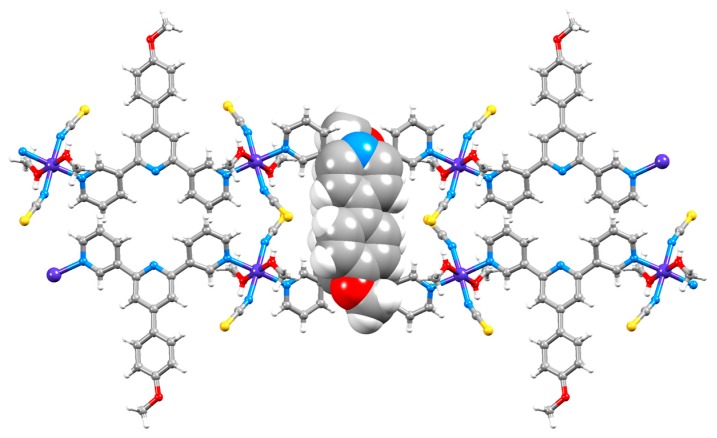
Packing of chains in [Co(**2**)(NCS)_2_(MeOH)_2_]*_n_*. View down the *b*-axis showing head-to-tail π-stacking between 4-methoxyphenylpyridine units of pairs of ligands **2**.

**Figure 6:**
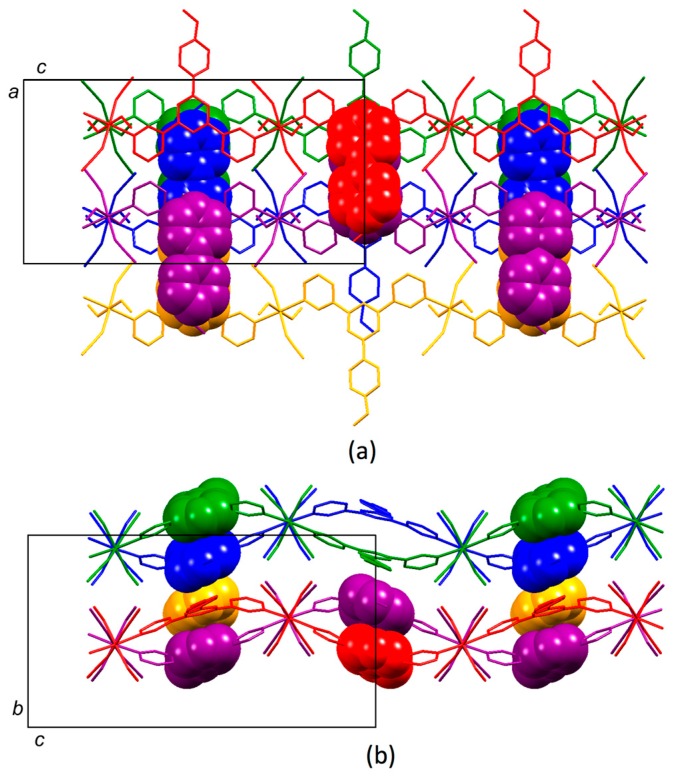
Packing of chains in [Co(**2**)(NCS)_2_(MeOH)_2_]*_n_*. π-Stacking between 4-methoxyphenylpyridine units in four adjacent chains (each depicted in a different colour) is shown, with the same part of the lattice viewed down (**a**) the *b*-axis and (**b**) the *a*-axis.

The 1D-chains in [Co(**2**)(NCS)_2_(MeOH)_2_]*_n_* approximately follow the crystallographic *c*-axis. Figure 5 shows a view down the *b*-axis. Adjacent chains associate through head-to-tail stacking of 4-methoxyphenylpyridine units (Figure 5). Although the inter-plane angle between stacked rings is 12.4°, the centroid_pyridine_-to-plane_phenyl_ and centroid-to-centroid distances are 3.25 and 3.64 Å, respectively, making these efficient interactions [54]. As Figure 5 shows, every other ligand in a given chain is oriented to opposite sides of the chain, and face-to-face π-stacking occurs between alternate pairs of ligands in adjacent chains as depicted for the blue/green and yellow/purple-coloured stacked units in Figure 6. The alternate stacking of the purple-coloured chain in Figure 6 with red- and orange-coloured chains leads to extended π-stacking which interlock the chains through the lattice.

### 3.4. Structures of [{Co(**2**)_2_(NCS)_2_}·3MeOH]_n_ and [{Co(**2**)_2_(NCS)_2_}·2.2CHCl_3_]_n_

**Figure 7:**
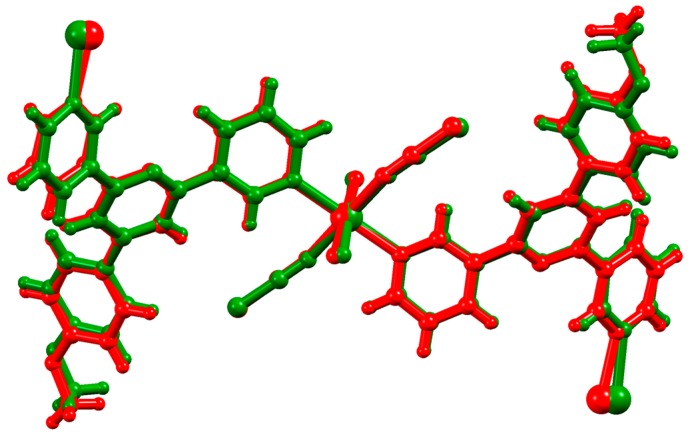
Overlay of the repeating units (with symmetry generated atoms) in [{Co(**2**)_2_(NCS)_2_}·3MeOH]*_n_* (shown in red) and [{Co(**2**)_2_(NCS)_2_}·2.2CHCl_3_]*_n_* (in green). Solvent molecules omitted. The angles between the planes of connected pyridine rings are 32.9° and 32.0° in [{Co(**2**)_2_(NCS)_2_}·3MeOH]*_n_* and 33.8° and 38.2° in [{Co(**2**)_2_(NCS)_2_}·2.2CHCl_3_]*_n_*; the angle between the rings in the 4-methoxyphenylpyridine unit is 50.8° in [{Co(**2**)_2_(NCS)_2_}·3MeOH]*_n_* and 50.5° in [{Co(**2**)_2_(NCS)_2_}·2.2CHCl_3_]*_n_*.

[{Co(**2**)_2_(NCS)_2_}·3MeOH]*_n_* and [{Co(**2**)_2_(NCS)_2_}·2.2CHCl_3_]*_n_* crystallize in the monclinic space groups *P*2_1_/*n* and *P*2_1_/*c*, respectively and are pseudopolymorphs. We discuss only the structure of [{Co(**2**)_2_(NCS)_2_}·3MeOH]*_n_* in detail, since the networks in the two structures are essentially the same. Figure 7 shows an overlay of the repeating units (with symmetry generated atoms) in the two structures and angles between the ring planes given in the caption confirm the similarity of the structures. The six-coordinated cobalt atom lies on an inversion centre.

**Figure 8:**
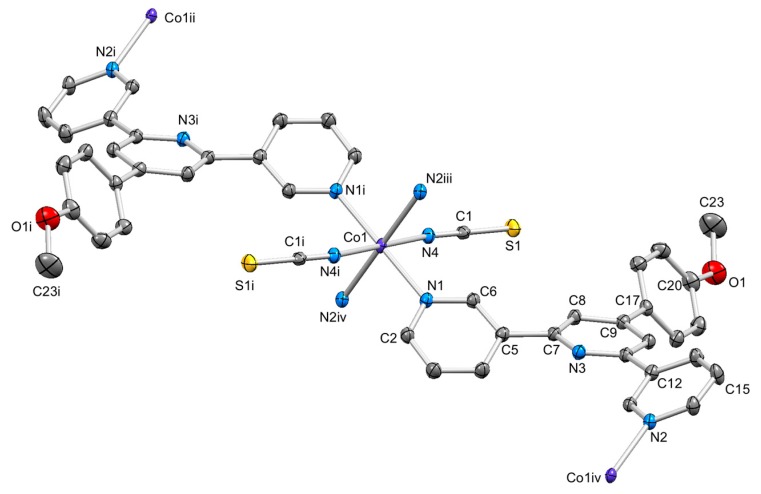
The repeating unit in the 2D network in [{Co(**2**)_2_(NCS)_2_}·3MeOH]*_n_* with symmetry generated atoms; ellipsoids are plotted at a 40% probability level, and H atoms omitted for clarity. Symmetry codes: i = 1–*x*, 1–*y*, –*z*; ii = ^1^/_2_–*x*, ^1^/_2_+*y*, –^1^/_2_–*z*; iii = –^1^/_2_+*x*, ^1^/_2_–*y*, –^1^/_2_+*z*; iv = ^3^/_2_–*x*, ^1^/_2_+*y*, ^1^/_2_–*z*. Selected bond distances: Co1–N4 2.0884(16), Co1–N2^iii^ = 2.2096(16), Co1–N1 = 2.1714(16), O1–C20 = 1.366(3), O1–C23 = 1.425(4) Å; C20–O1–C23 = 117.3(2)°, by symmetry, N1–Co1–N1^i^ = N2^iii^–Co1–N2^iv^ = N4–Co1–N4^i^ =180.0°.

**Figure 9:**
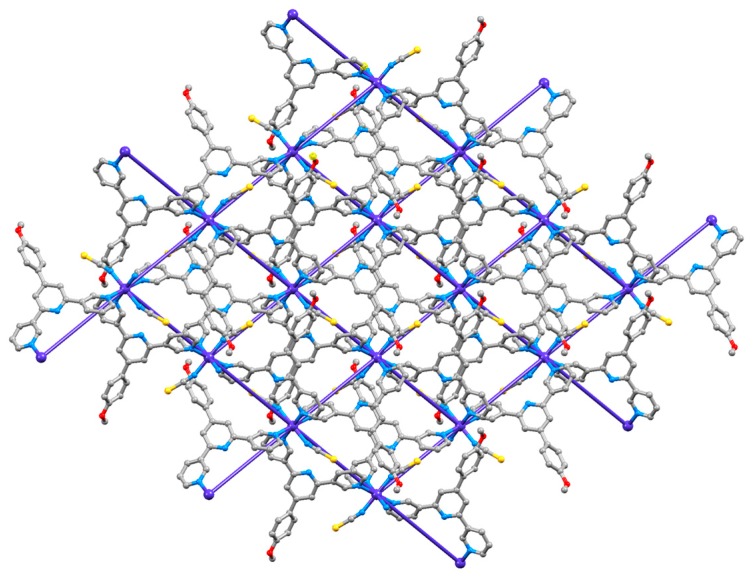
Part of one 2D network of [Co(**2**)_2_(NCS)_2_(MeOH)_2_]*_n_* showing the 4-connecting Co nodes in the (4,4) net.

**Figure 10:**
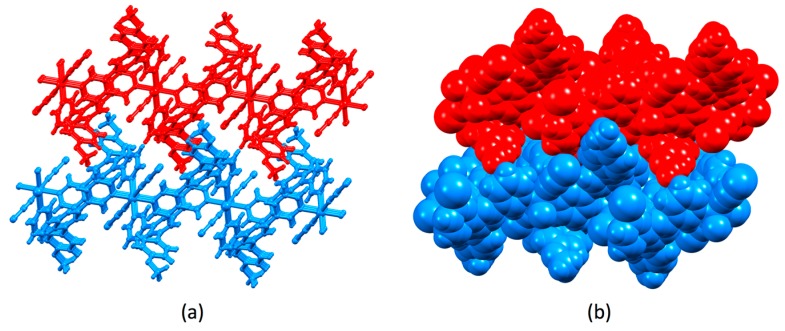
Parts of two adjacent 2D sheets in [Co(**2**)_2_(NCS)_2_(MeOH)_2_]*_n_*: (**a**) ball-and-stick and (**b**) space-filling representations.

The repeating unit in [{Co(**2**)_2_(NCS)_2_}·3MeOH]*_n_* (with symmetry generated atoms) is displayed in Figure 8, and the figure caption gives important bond parameters. All bond distances are as expected. The coordination mode of **2** mimics that in [Co(**2**)(NCS)_2_(MeOH)_2_]*_n_*. Atom Co1 acts as a 4-connecting node and [{Co(**2**)_2_(NCS)_2_}·3MeOH]*_n_* is a (4,4) net with the organic ligands acting as linkers (Figure 9). In contrast to the conformation II adopted by **2** in [Co(**2**)(NCS)_2_(MeOH)_2_]*_n_* (see Section 3.2), ligand **2** in [{Co(**2**)_2_(NCS)_2_}·3MeOH]*_n_* adopts a conformation similar to Mode III in Scheme 1. The twist angles between the pyridine rings with N1/N3 and N3/N2 are 32.9° and 32.0°, and that between the rings in the 4-methoxyphenylpyridine unit is 50.8°. The latter is significantly greater than the corresponding angle in [Co(**2**)(NCS)_2_(MeOH)_2_]*_n_* (12.4°) and reflects the fact that in the 2D-net, ligand **2** does not engage in π-stacking interactions. As Figure 10 shows, the 4-methoxyphenyl units protrude from the upper and lower sides of each (4,4) net and are accommodated in pockets in an adjacent sheet. The closest C–H**^...^**π contact between sheets is 3.55 Å (CH_Me_**^...^**centroid_pyridine_) which is rather long to be significant [55].

In [{Co(**2**)_2_(NCS)_2_}·3MeOH]*_n_*, SQUEEZE had to be applied to the solvent region, whereas is [{Co(**2**)_2_(NCS)_2_}·2.2CHCl_3_]*_n_*, the solvent was modelled as one CHCl_3_ molecule disordered around an inversion centre and one CHCl_3_ disordered over 2 orientations. The CHCl_3_ molecules occupy sites both within and between the 2D sheets, and presumably MeOH molecules in [{Co(**2**)_2_(NCS)_2_}·3MeOH]*_n_* occupy analogous sites. 

## 4. Conclusions

We have described the assembly of 1D-coordination polymers or 2D-networks from reactions of Co(NCS)_2_ with ligand **2**. In a series of experiments, crystals were grown at room temperature by layering a MeOH solution of Co(NCS)_2_ over a CHCl_3_ solution of **2**. With a Co(NCS)_2_ to **2** molar ratio of 1:1 or 2:1, the product was [Co(**2**)(NCS)_2_(MeOH)_2_]*_n_* consisting of 1D-chains with coordinated methanol. Powder XRD confirmed that the crystals selected for single-crystal X-ray diffraction or cell dimension checks were representative of the bulk sample. Longer crystallization times with a Co(NCS)_2_:**2** molar ratio of 1:1 yielded crystals of [Co(**2**)(NCS)_2_(MeOH)_2_]*_n_* (1D-chain) and the pseudopolymorphs [{Co(**2**)_2_(NCS)_2_}·3MeOH]*_n_* and [{Co(**2**)_2_(NCS)_2_}·2.2CHCl_3_]*_n_* (2D-nets), each type of crystal originating from a different zone in the crystallization tube. PXRD for this last experiment confirms that the dominant product in the bulk sample was the 1D-coordination polymer. This investigation highlights the interplay between thermodynamic and kinetic products from crystallization processes, and illustrates once again [56] that we are far from fully understanding the assembly algorithms of metallo-coordination polymers involving terpyridine ligands. In particular, these experiments emphasize that, although the crystalline products represent local thermodynamic minima, the growth process is far from equilibrium and the local concentrations and ratios of reactants are time dependent. 

## Supplementary Materials

The following are available online at https://www.mdpi.com/2073-4360/11/7/1224/s1, Figure S1 Electrospray mass spectrum of **2**; Figures S2–S5: NMR spectra of **2**; Figures S6–S9: powder X-ray diffraction data.
