# Correction: Rocco, D.; et al. Competition in Coordination Assemblies: 1D-Coordination Polymer or 2D-Nets Based on Co(NCS)_2_ and 4′-(4-methoxy)-3,2′:6′,3″-terpyridine. *Polymers* 2019, *11*, 1224; doi:10.3390/polym11071224

**DOI:** 10.3390/polym11101537

**Published:** 2019-09-20

**Authors:** Dalila Rocco, Alessandro Prescimone, Y. Maximilian Klein, Dariusz J. Gawryluk, Edwin C. Constable, Catherine E. Housecroft

**Affiliations:** 1 Department of Chemistry, University of Basel, BPR 1096, Mattenstrasse 24a, CH-4058 Basel, Switzerland; dalila.rocco@unibas.ch (D.R.); alessandro.prescimone@uibas.ch (A.P.); edwin.constable@unibas.ch (E.C.C.); 2 Laboratory for Multiscale Materials Experiments, Paul Scherrer Institut, CH-5232 Villigen PSI, Switzerland; maximilian.klein@psi.ch (Y.M.K.); dariusz.gawryluk@psi.ch (D.J.G.)

We wish to make the following corrections to our published paper [1]. The formulae of the coordination networks in the captions to Figures 9 and 10 were incorrectly written. The authors apologize for the error. 

The correct captions should be:

**Figure 9**. Part of one 2D network of [{Co(**2**)_2_(NCS)_2_}·3MeOH]*_n_* showing the 4-connecting Co nodes in the (4,4) net.
**Figure 10**. Parts of two adjacent 2D sheets in [{Co(**2**)_2_(NCS)_2_}·3MeOH]*_n_*: (a) ball-and-stick and (b) space-filling representations.
