# Supplementary material for: Competition in Coordination Assemblies: 1D-Coordination Polymer or 2D-Nets Based on Co(NCS)2 and 4′-(4-methoxyphenyl)-3,2′:6′,3″-terpyridine
Source: Polymers (Basel). 2019 Jul 23;11(7):1224. doi: 10.3390/polym11071224 (PMC6680941; doi:10.3390/polym11071224)
Supplement: Supplementary file 1 [file polymers-11-01224-s001.pdf]

Supporting Information

Competition in Coordination Assemblies: 1D-Coordination Polymer or 2D-Nets Based on Co(NCS)2 and 4′-(4-methoxyphenyl)-3,2′:6′,3″-terpyridine

Dalila Rocco, [1] Alessandro Prescimone, [1] Y. Maximilian Klein, [2] Dariusz J. Gawryluk, [2] Edwin C. Constable [1] and Catherine E. Housecroft [1]*

[1] Department of Chemistry, University of Basel, BPR 1096, Mattenstrasse 24a, CH-4058 Basel, Switzerland
[2] Laboratory for Multiscale Materials Experiments, Paul Scherrer Institut, CH-5232 Villigen PSI, Switzerland
* Correspondence: catherine.housecroft@unibas.ch

ORCID C.E.H. 0000-0002-8074-0089; E.C.C. 0000-0003-4916-4041

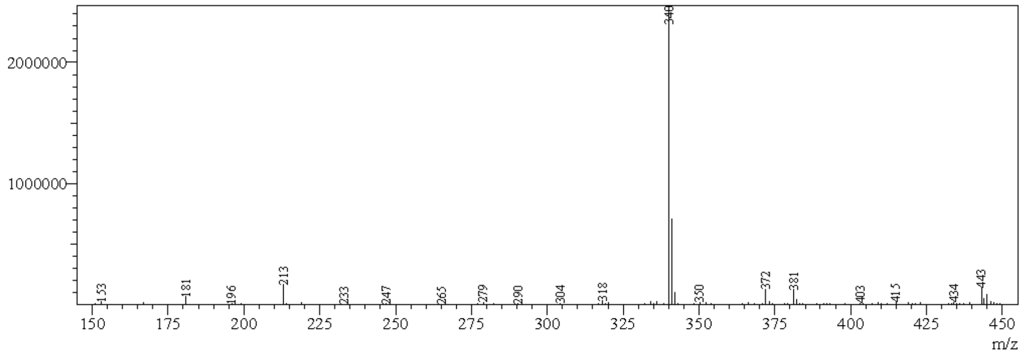

Figure S1. Electrospray mass spectrum of **2**.

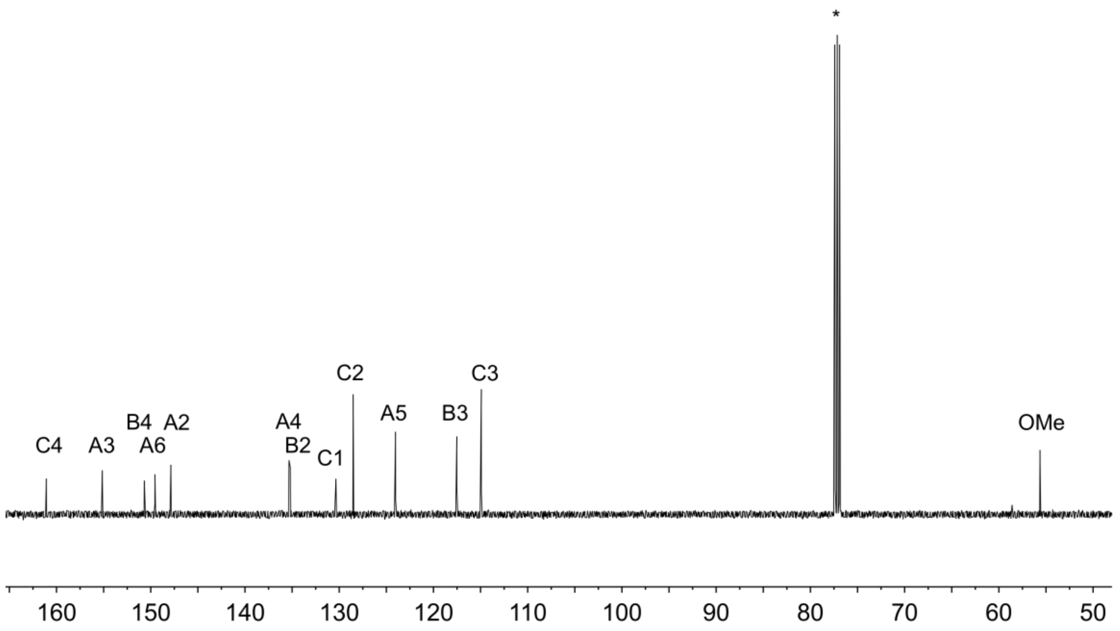

Figure S2. 126 MHz $^{13}C\{^{1}H\}$ NMR spectrum of **2** ($CDCl_3$, 298 K). * = $CDCl_3$. See Scheme 2 for atom labelling.

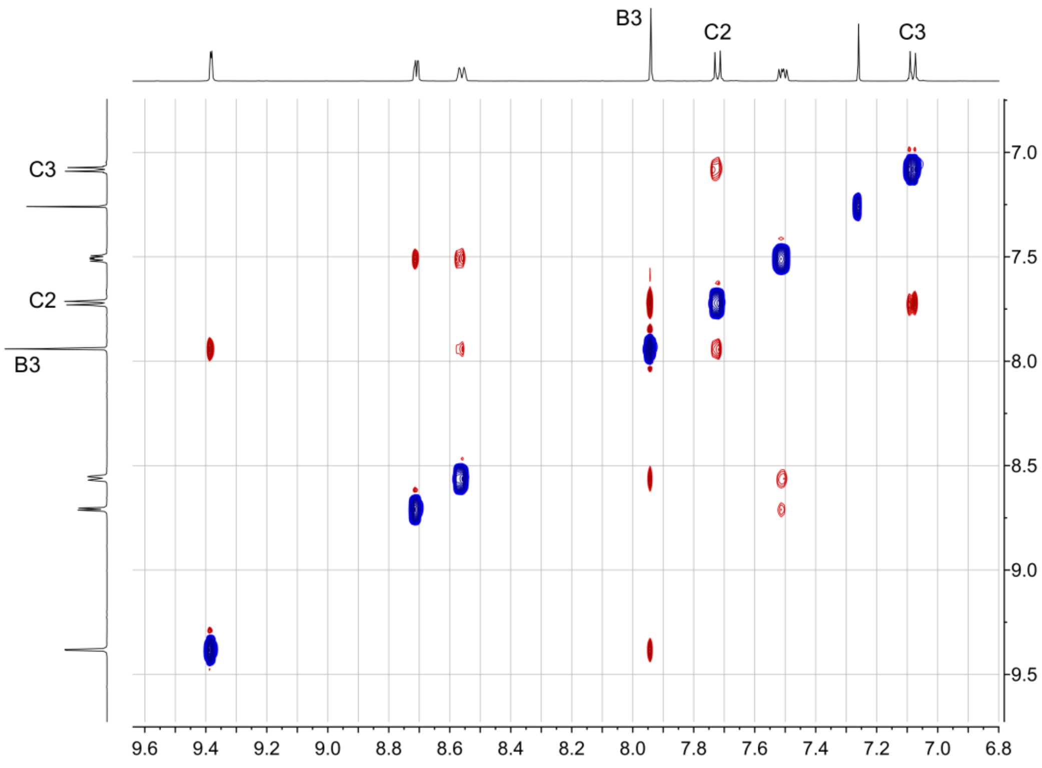

Figure S3. NOESY spectrum of **2** (500 MHz ^{1}H NMR, $CDCl_3$, 298 K).

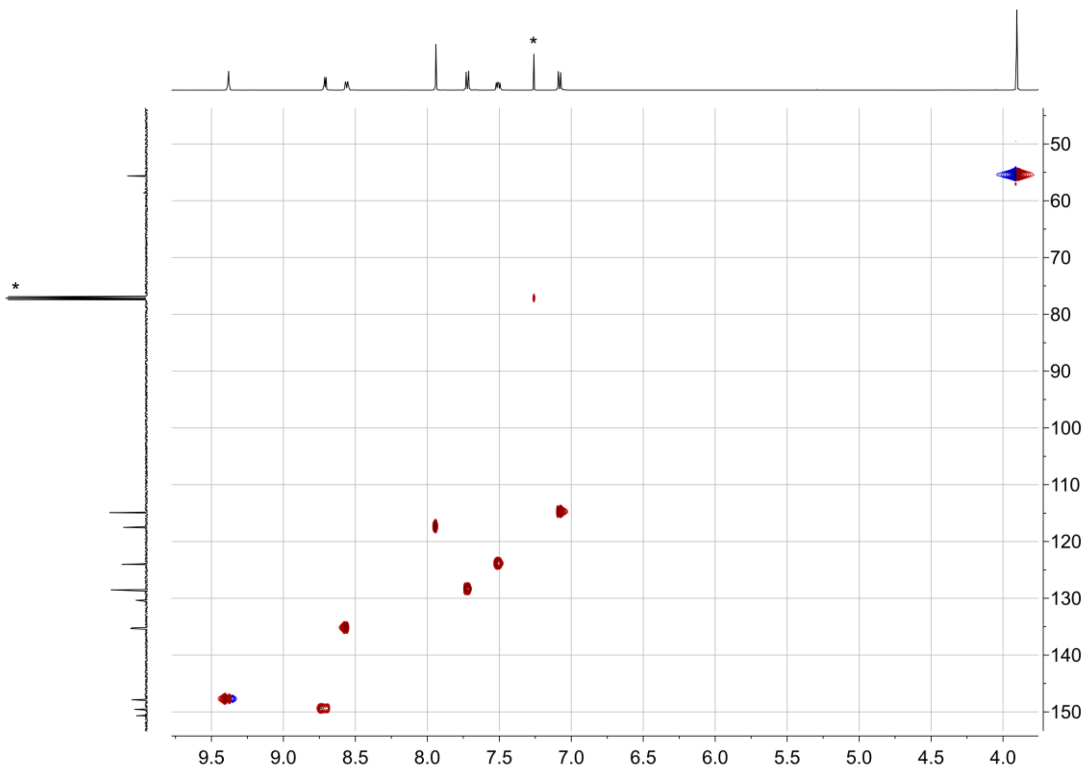

Figure S4. HMQC spectrum of **2** (500 MHz ^{1}H, 126 MHz $^{13}C\{^{1}H\}$, $CDCl_3$, 298 K).

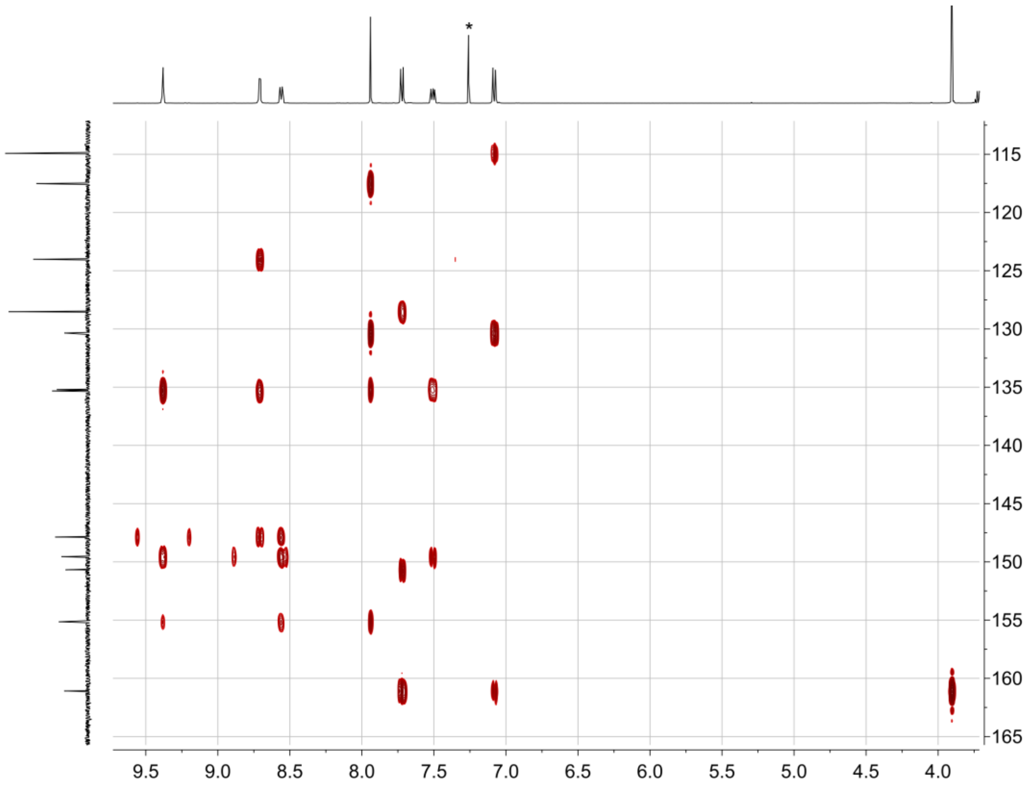

Figure S5. HMBC spectrum of **2** (500 MHz ^{1}H, 126 MHz $^{13}C\{^{1}H\}$, $CDCl_3$, 298 K).

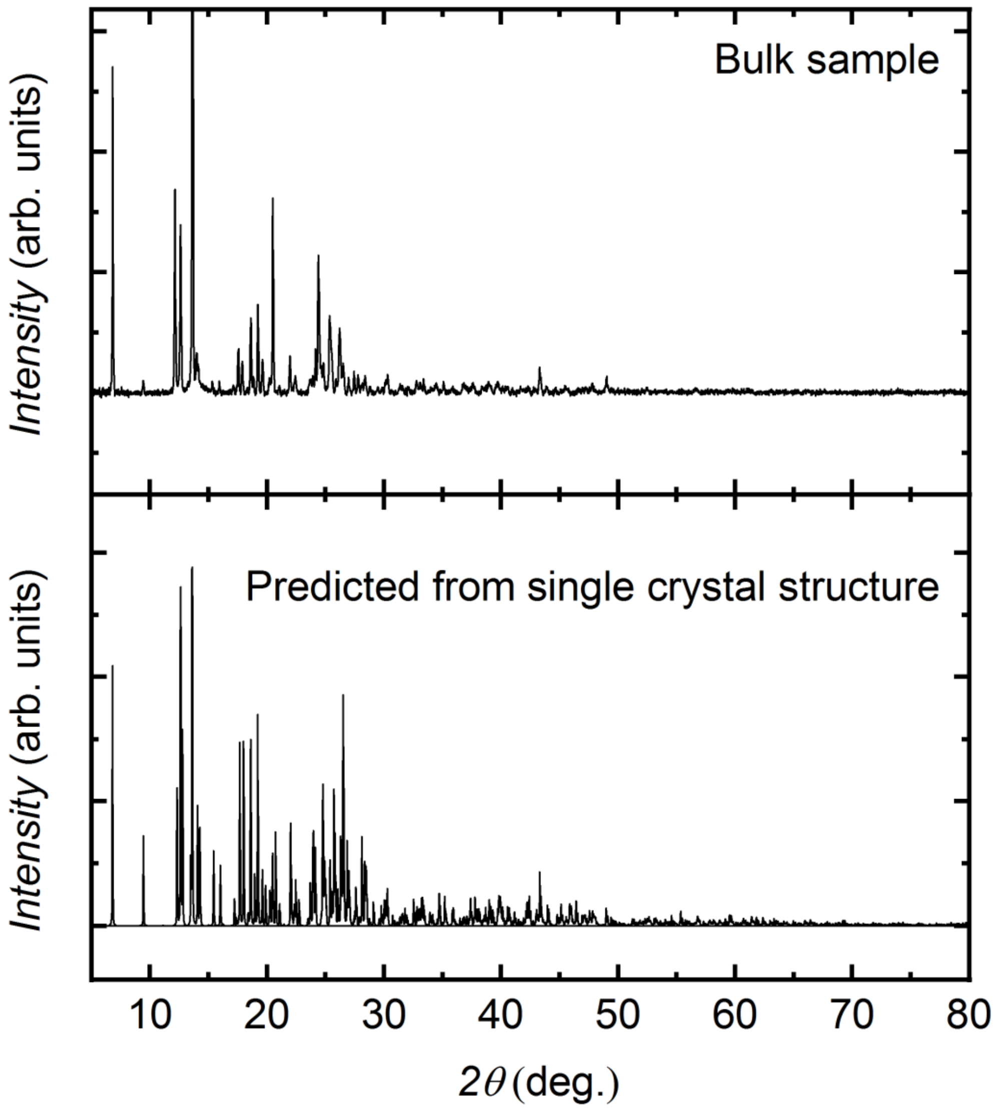

Figure S6. Powder XRD pattern (295 K) of the bulk material from experiment I compared to that predicted from the single crystal structure (130 K) of $[Co(\mathbf{2})(NCS)_2(MeOH)_2]_n$.

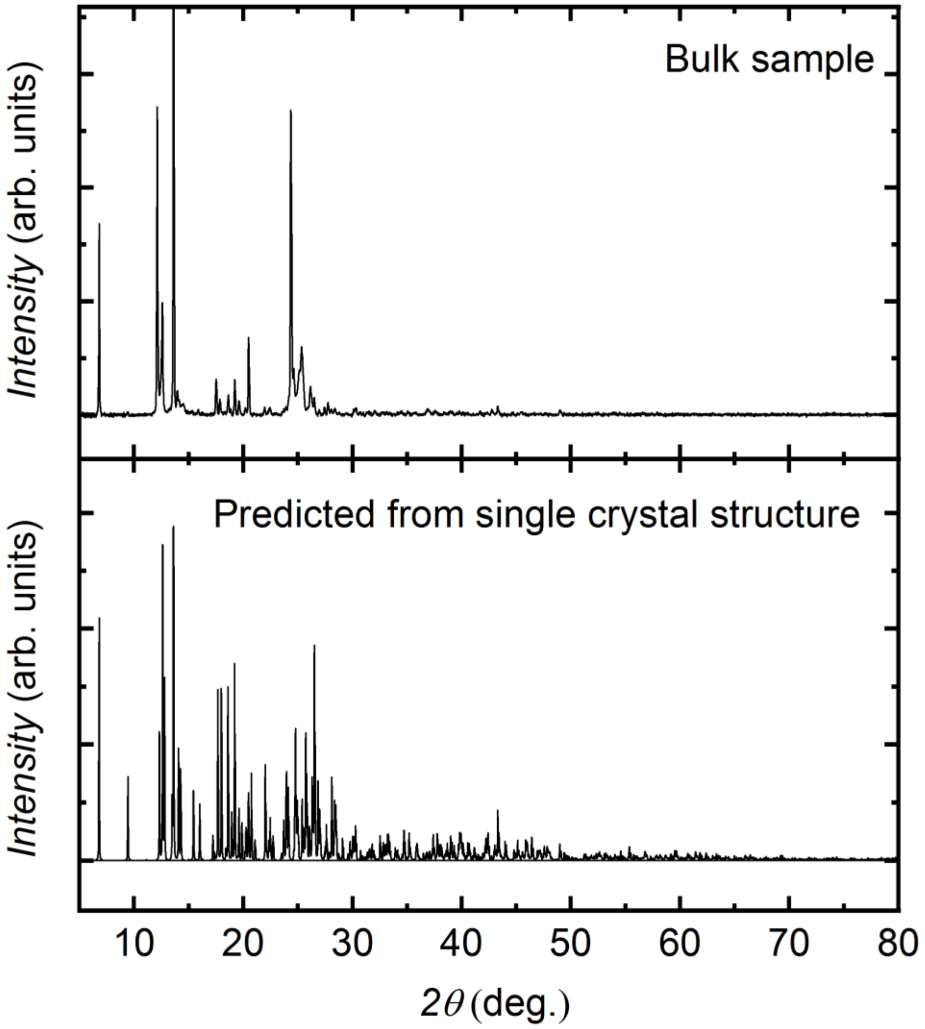

Figure S7. Powder XRD pattern (295 K) of the bulk material from experiment III compared to that predicted from the single crystal structure (130 K) of $[Co(\mathbf{2})(NCS)_2(MeOH)_2]_n$.

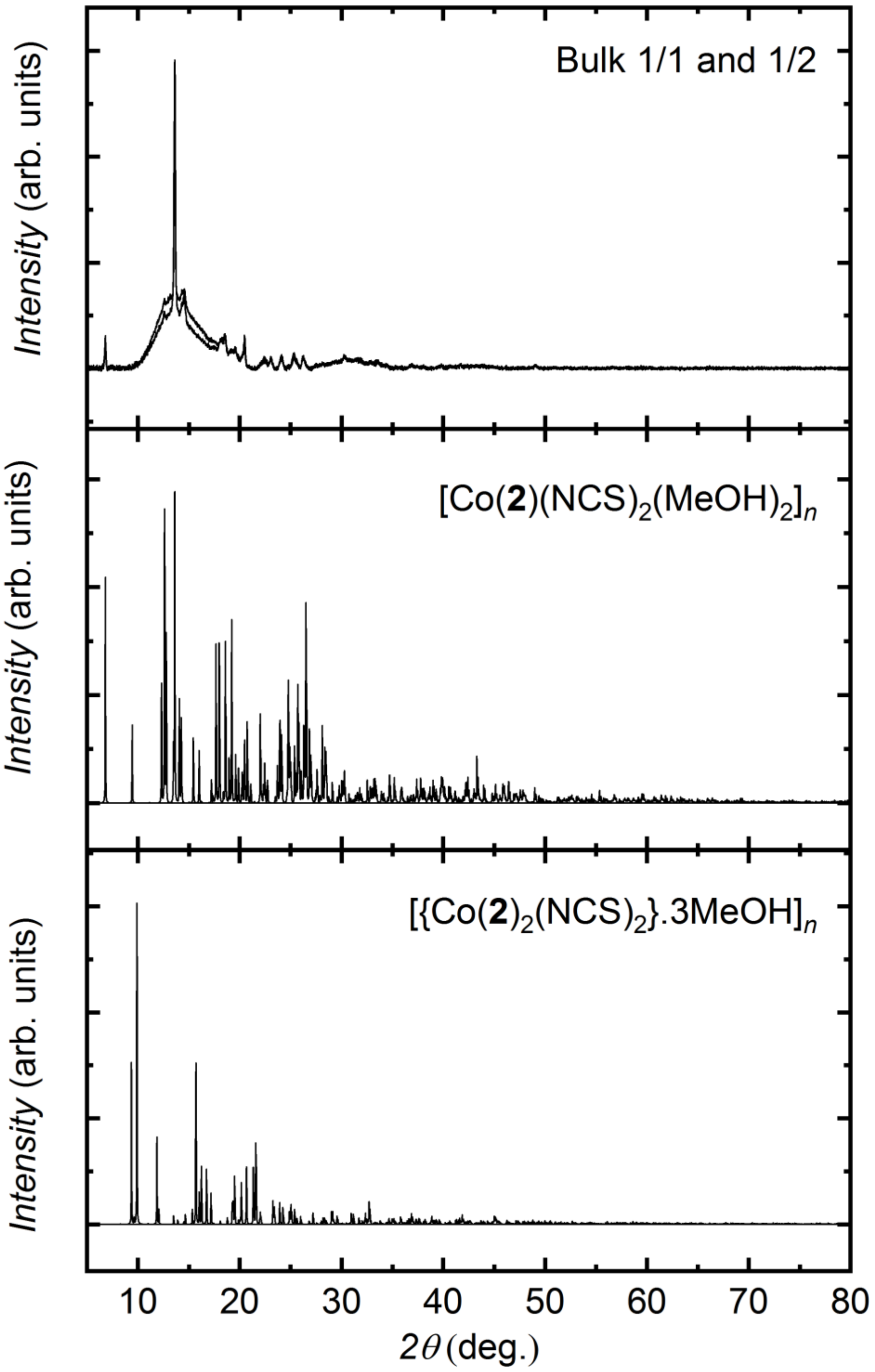

Figure S8. Top: Overlay of the PXRD patterns from the powders obtained from the 1 : 1 and 1 : 2 bulk reactions of $Co(NCS)_2$ and **2**. Middle and lower figures: Predicted powder patterns from the single crystal structures of $[Co(\mathbf{2})(NCS)_2(MeOH)_2]_n$ and $[\{Co(\mathbf{2})_2(NCS)_2\}{\cdot}3MeOH]_n$.

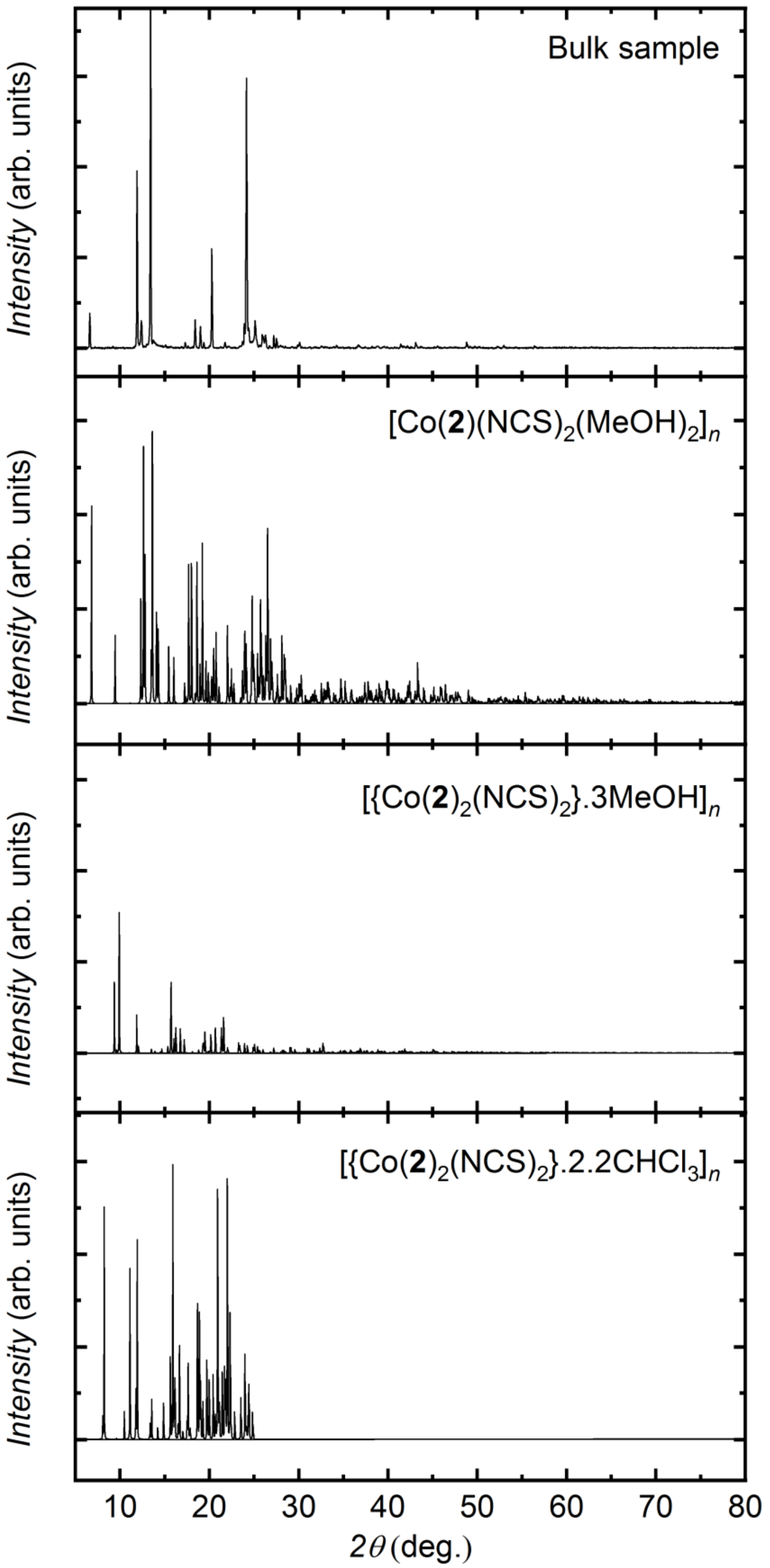

Figure S9. Powder XRD pattern (295 K) of the bulk material from experiment II compared to those predicted from the single crystal structures (130 K) of $[Co(\mathbf{2})(NCS)_2(MeOH)_2]_n$, $[\{Co(\mathbf{2})_2(NCS)_2\}\cdot 3MeOH]_n$ and $[\{Co(\mathbf{2})_2(NCS)_2\}\cdot 2.2CHCl_3]_n$.
